# MEMS-Based Pulse Wave Sensor Utilizing a Piezoresistive Cantilever

**DOI:** 10.3390/s20041052

**Published:** 2020-02-15

**Authors:** Thanh-Vinh Nguyen, Yuya Mizuki, Takuya Tsukagoshi, Tomoyuki Takahata, Masaaki Ichiki, Isao Shimoyama

**Affiliations:** 1 Sensing System Research Center, National Institute of Advanced Industrial Science and Technology (AIST), Ibaraki 305-8564, Japan; ichiki-m@aist.go.jp; 2 Graduate School of Information Science and Technology, The University of Tokyo, Tokyo 113-8656, Japan; mizuki@hybrid.t.u-tokyo.ac.jp (Y.M.); takahata@mi.t.u-tokyo.ac.jp (T.T.); 3 Department of Intelligent Robotics, Toyama Prefectural University, Toyama 939-0398, Japan; tsukagoshi@pu-toyama.ac.jp (T.T.); i-shimoyama@pu-toyama.ac.jp (I.S.)

**Keywords:** pulse wave, pulse wave velocity, MEMS, piezoresistive, cantilever

## Abstract

This paper reports on a microelectromechanical systems (MEMS)-based sensor for pulse wave measurement. The sensor consists of an air chamber with a thin membrane and a 300-nm thick piezoresistive cantilever placed inside the chamber. When the membrane of the chamber is in contact with the skin above a vessel of a subject, the pulse wave of the subject causes the membrane to deform, leading to a change in the chamber pressure. This pressure change results in bending of the cantilever and change in the resistance of the cantilever, hence the pulse wave of the subject can be measured by monitoring the resistance of the cantilever. In this paper, we report the sensor design and fabrication, and demonstrate the measurement of the pulse wave using the fabricated sensor. Finally, measurement of the pulse wave velocity (PWV) is demonstrated by simultaneously measuring pulse waves at two points using the two fabricated sensor devices. Furthermore, the effect of breath holding on PWV is investigated. We showed that the proposed sensor can be used to continuously measure the PWV for each pulse, which indicates the possibility of using the sensor for continuous blood pressure measurement.

## 1. Introduction

Pulse wave is one of the four fundamental vital signs—body temperature, pulse wave, blood pressure, and respiration rate. It is possible to obtain some vital pieces of information related to the health condition of a subject as well as to predict the development of diseases from the pulse wave of the subject [1]. For example, one can derive the pulse wave velocity by measuring the pulse waves at two points on a vessel, which is a useful indicator of hypertension [2,3], arteriosclerosis [4,5], and diabetes [6,7]. Moreover, previous studies have also demonstrated the relationship between the pulse wave velocity (PWV) and blood pressure [8,9], which indicates the possibility of realizing a wearable cuff-less sensor for continuous measurement of blood pressure by measuring the PWV using two wearable pulse wave sensors.

Many methods have been proposed in previous studies to measure the pulse wave; these methods can be roughly divided into two categories based on their measurement principle, namely optics-based and force sensing-based methods. The most common device used in the optics-based method is the pulse oximeter, which is based on photoplethysmography [10,11]. A pulse oximeter detects pulse waves by monitoring the volume change of the blood vessel using an optical system consisting of an LED and a photodiode. This method has the advantage of simple measurement and has been widely used, for example, in smartwatches to estimate the heart rate of the wearers. However, this method has several limitations including high power consumption compared with the force sensing-based method and the non-negligible effect of the skin tone and skin pigmentation on the measurement result [12,13]. In the force-sensing based method, the pulse wave is measured by measuring the pulse-induced deformation of the skin above the vessel using a force sensor or a strain sensor. These force/strain sensors can be divided into two categories based on the type of material, namely polymer-based [14,15,16,17] and Si-based [18,19,20] sensors. Polymer-based sensors utilize soft materials, such as a thin elastic film, as the sensor substrate and hence have high flexibility and are suitable for wearable sensors. On the other hand, although Si-based sensors are not flexible, they have some interesting advantages including good functionalities (e.g., fast response and low hysteresis) and can be easily mass produced. In conventional Si-based sensors, the force sensing elements, which are usually fragile, should be embedded in an elastic body so that the sensor can be pushed against the human skin. However, this approach is often a trade-off between the sensitivity and the durability of the sensors; that is, if the Young’s modulus of the elastic body decreases, the sensitivity will increase but the sensor can be easily broken under static load.

**Figure 1:**
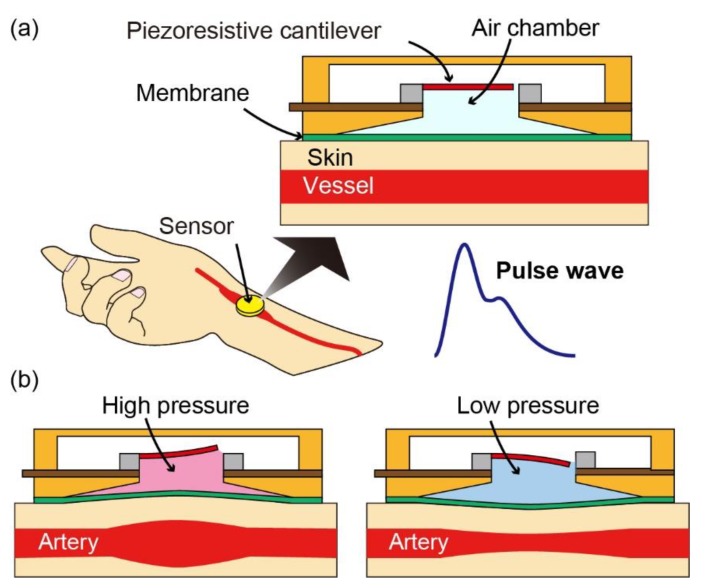
(**a**) Conceptual schematic diagram of the proposed sensor to measure pulse wave. The proposed sensor consists of a piezoresistive cantilever and a chamber covered with a soft membrane. (**b**) Sensing principle of the proposed sensor.

In this study, we propose an Si-based pulse wave sensor to achieve both excellent sensitivity and robustness. The conceptual sketch of the proposed sensor is shown in Figure 1a. The sensor consists of an air chamber covered with a thin and soft membrane and a piezoresistive cantilever placed inside the chamber. When the membrane of the sensor is placed on the vessel, deformation of the skin above the vessel causes the membrane to deform, resulting in a change in the air pressure of the chamber (Figure 1b). The pulse wave of the subject can be measured by monitoring the resistance of the cantilever as variations in the chamber pressure result in bending of the cantilever and change in the resistance. This structure of the air chamber and thin membrane was utilized in our previous research to measure the vibration of small droplets [21]. When a static load is applied, the cantilever will initially bend as a result of the pressure difference. However, the cantilever will return to its original position as the chamber pressure will eventually balance the pressure outside the chamber owing to the gaps surrounding the cantilever, allowing the air inside the chamber to escape. Therefore, damage to the cantilever can be prevented even when a large static load is applied to the sensor. This ensures that the cantilever can be made very thin (200 nm-thick in this study) to achieve high sensitivity without being damaged by the static force. In other words, the sensor design in this study achieved both high robustness to static load and high sensitivity to dynamic load. The comparison of the proposed sensor in this paper and those in previous studies is shown in Table 1. The proposed sensor has no effect by the skin color which is an advantage in comparison with photoplethysmography-based sensors. In comparison with polymer-based sensors, the proposed sensor is advantageous in term of mass-production compatibility. Moreover, in comparison with previous MEMS-based sensors in which, the sensing element is embedded in elastomer, gel or liquid, the proposed sensor is advantageous in term of sensitivity and robustness. In this paper, we report the design, fabrication, and evaluation of the proposed sensor. Measurements of the pulse wave and PWV using the fabricated sensors are also presented.

## 2. Materials and Methods

### 2.1. Piezoresistive Cantilever

**Figure 2:**
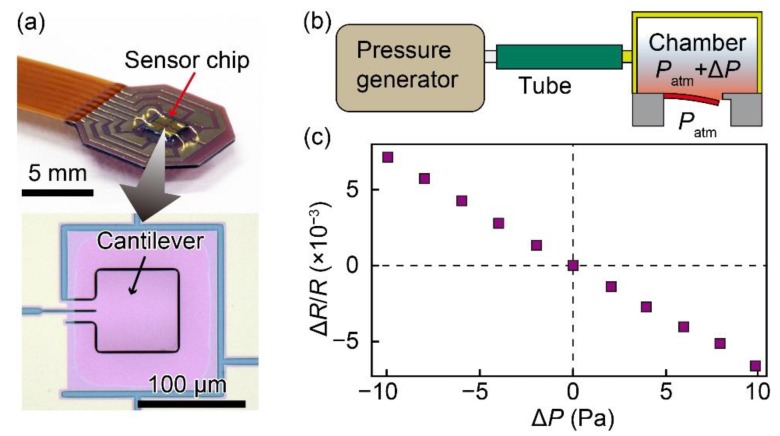
(**a**) Photographs of the fabricated sensor chip attached on a flexible substrate and the cantilever. (**b,c**) Conceptual sketch of the calibration setup and the calibration result.

In this study, a piezoresistive cantilever was used as the pressure sensing element. Different from the cantilevers used for force measurement, such as those used in atomic force microscopes, the cantilever in this study was designed to measure the differential air pressures applied at the top and the back sides of the cantilever. In the sensor design, the cantilever was surrounded by a narrow gap of approximately 1 μm to significantly reduce air leak through the gap and ensure that the cantilever can measure the differential pressure at frequencies as low as 0.1 Hz [22,23]. The cantilever consists of a pad with dimensions of 80 μm × 80 μm and two hinges with dimensions of 30 μm × 10 μm. The thickness of the cantilever is 200 nm. Two electrodes were placed at the roots of the two hinges and the resistance of the cantilever is defined as the resistance between the two electrodes. The fabrication process of the cantilever can be found in previous studies [24,25,26,27,28,29]. A photograph of the fabricated sensor chip attached and wire-bonded to a flexible substrate and a photograph of the cantilever are shown in Figure 2a. The dimensions of the sensor chip are 1.5 mm × 1.5 mm × 0.3 mm and the thickness of the flexible substrate is 0.2 mm.

The fabricated cantilever was calibrated using the setup illustrated in Figure 2b. A differential pressure in the range of ±10 Pa was applied to the cantilever using a pressure generator. The fractional resistance change of the cantilever was measured using the setup reported in previous studies [24,25,26,27,28,29,30,31,32], which consists of the Wheatstone bridge circuit and an amplifying circuit whose output is recorded using a ScopeCorder (Yokogawa Test & Measurement Co., Tokyo, Japan, DL850). The calibration result in Figure 2c shows the linear relationship between the differential pressure and the fractional resistance change of the cantilever. In other words, the differential pressure applied on the cantilever can be measured by measuring its resistance change. Moreover, from the calibration result and the noise level of the measurement setup, which was approximately 20 mV, the resolution of the sensor can be estimated to be approximately 0.02 Pa.

### 2.2. Sensor Device

**Figure 3:**
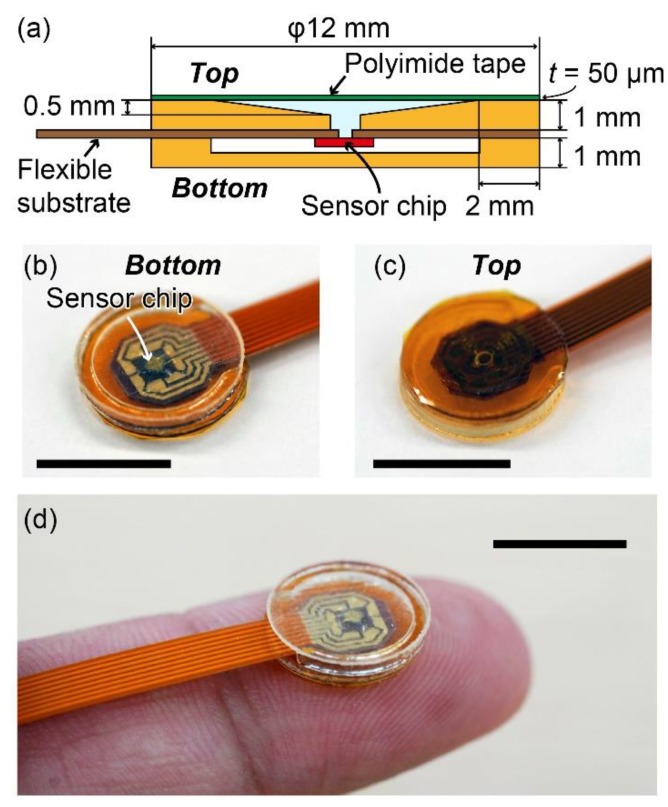
(**a**) Structure of the sensor. (**b**,**c**) Photographs of the bottom and top surfaces of the fabricated sensor. (**d**) Photograph of the sensor placed on an index finger. The scale bars in (**b**–**d**) are 1 cm.

We assembled the sensor device for pulse wave measurement using the fabricated sensor chip. The structure of the sensor device is shown in Figure 3a. The flexible substrate on which the sensor chip was attached was sandwiched by two caps: one cap covered the sensor chip to protect it and the other cap has a flexible membrane (50 μm-thick polyimide tape) as the sensor head to detect the pulse wave. The cap with the flexible membrane has a through hole connected to the hole of the sensor chip such that the pressure inside the chamber of the cap can be detected by the sensor. The caps were fabricated using a 3D printer and the thickness of each cap is 1 mm. In principle, a large membrane area is desired to increase the sensitivity of the sensor to pulse wave. However, in this study, the sensor was designed to measure pulse wave when attached to a fingertip; therefore, the diameter of the cap of the sensor was designed to be 12 mm, which is slightly smaller than the size of an index finger of adults. Photographs of the assembled sensor device are shown in Figure 3b,c. A photograph of the sensor device placed on a finger is shown in Figure 3d.

## 3. Results and Discussion

### 3.1. Measurement of Pulse Wave

**Figure 4:**
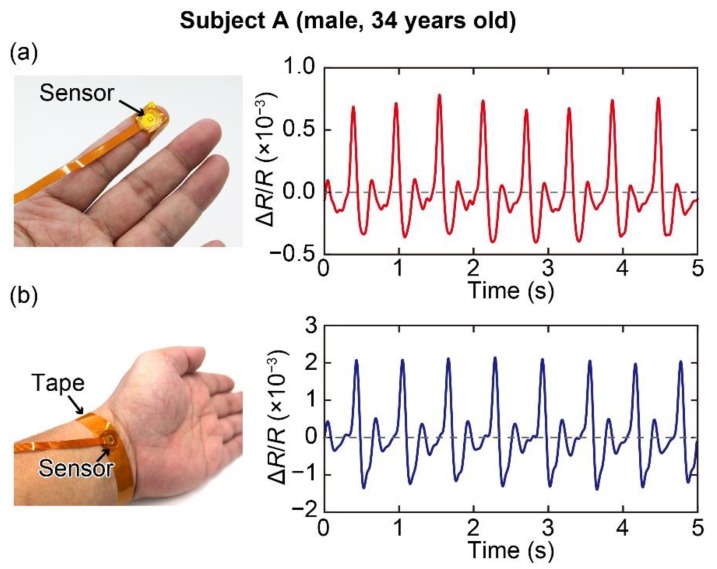
(**a**) Measurement of pulse wave at the tip of the subject’s index finger. (**b**) Measurement of pulse wave at the wrist of the subject.

First, we measured the pulse waves using the fabricated sensor device. The measurements were conducted for 3 healthy subjects: subject A (male, 34 years old), subject B (male, 42 years old), and subject C (male, 31 years old). All subjects gave their informed consent for inclusion before they participated in the study. The study was conducted in accordance with the Declaration of Helsinki, and the protocol was approved by the Ethics Committee of National Institute of Advanced Industrial Science and Technology (AIST) (Project code: 2019-998). In the measurements, the sensor device was attached to the wrist or the tip of the subject’s index finger using a tape and the change in the resistance of the cantilever was measured at a sampling rate of 1000 samples/s and frequency band of 0–400 Hz using the same setup described in Section 2.1. The recorded signals were filtered using a low pass filter with a cut-off frequency of 10 Hz. The measurement results of subject A are shown in Figure 4. The pulse waves measured at the wrist and tip of the index finger for 5 s are shown in Figure 4a,b, respectively. The results show that pulse waves can be measured by the sensor and the pulse waves measured at the tip of the subject’s index finger and at the wrist have similar shapes. Moreover, the heart rates of the subject calculated from the measured pulse waves were approximately 103 and 97 beats per minute (bpm), respectively. The difference in the heart rates derived from the measured pulse waves is simply due to change in the heart rate with time.

**Figure 5:**
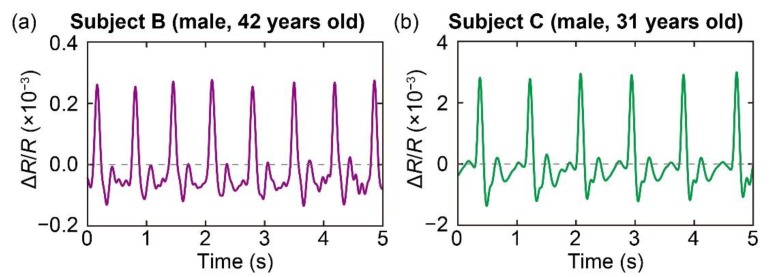
Measurement of pulse waves of (**a**) subject B and (**b**) subject C. The sensor was attached to the tip of the index fingers of the subjects.

The measurement results for subject B and subject C are shown in Figure 5a,b, respectively. For both subjects, the sensor was attached to their index fingers. It is clear that the sensor can measure the pulse waves of both subjects. The results also show the difference in the amplitudes of the subjects’ pulse waves; the amplitude of the pulse waves of subject C was 10 times higher than that of subject B. Moreover, the heart rates of the subjects were calculated as approximately 90 bpm and 69 bpm for subjects B and C, respectively.

### 3.2. Measurement of Pulse Wave Velocity

**Figure 6:**
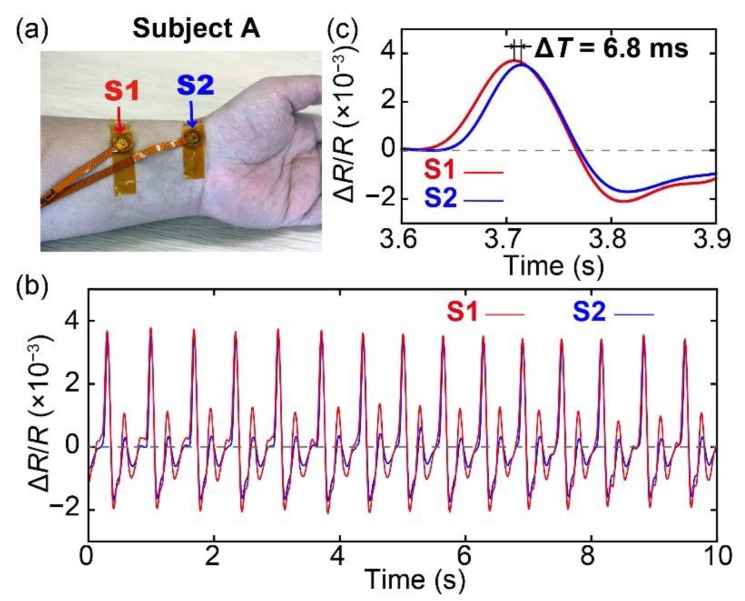
(**a**) Photograph of the two sensors placed at two points on the wrist of the subject to measure the pulse wave velocity. (**b**) Pulse waves measured using sensors S1 and S2. (**c**) Zoomed-in view of the pulse wave measured using the two sensors.

Next, we measured the pulse wave velocity using the proposed sensor. Two fabricated sensors, S1 and S2, were attached to two points on the wrist of subject A as shown in Figure 6a. The distance between the two sensors was approximately 50 mm. The measured pulse waves obtained from the outputs of the two sensors are shown in Figure 6b and a zoomed-in view of the graph is shown in Figure 6c. It is obvious that the peak of the pulse wave measured using S1 occurred earlier than that of the pulse wave measured using S2 because sensor S1 is relatively closer to the heart than sensor S2. The pulse transit time was calculated from the outputs of the sensors as the delay in the peaks of the two signals to be approximately 6.8 ms. Moreover, the pulse velocity calculated from the pulse transit time was approximately 7.4 m/s, which is in the range of a normal PWV reported in previous studies [33,34]. The result demonstrates that a wearable PWV sensor can be realized by integrating the two proposed sensors in a wrist band.

### 3.3. Effect of Breath Holding on PWV

**Figure 7:**
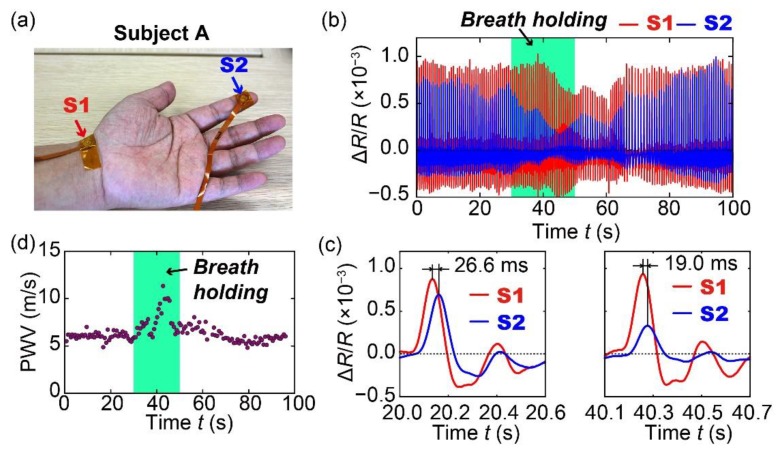
(**a**) Photograph of the sensors attached to the index finger and the wrist of subject A to measure the pulse wave velocity. (**b**) Pulse waves measured using the two sensors for 100 s. (**c**) Measured pulse waves before and during breath holding. (**d**) Measured PWV for 100 s. The results clearly show that breath holding can lead to increase in PWV.

In this section, continuous measurement of pulse waves using the fabricated sensors and the effect of breath holding on the PWV derived from the measurement result are presented. In the measurement, two sensors were attached to the wrist and the index finger of subject A as shown in Figure 7a. The distance between the two sensors was approximately 155 mm. The measurement of the pulse waves was carried out for 100 s, during which the subject was asked to hold his breath for 20 s (*t* = 30 s to 50 s) while breathing normally before and after this period. This experiment can be used to investigate the dependence of PWV on breath holding as previous studies reported that the blood pressure and PWV increase if one holds his or her breath [35,36]. The pulse waves measured for 100 s are shown in Figure 7b. Moreover, the zoomed-in views of the pulses at *t* = 20 s (before breath holding) and *t* = 40 s (during breath holding) are shown in Figure 7c. The results show that when the subject held his breath, the pulse transit time became shorter. Figure 7d shows the PWV calculated for all the pulses; it indicates that PWV gradually increased when the subject held his breath and the maximum value of PWV was more than 10 m/s, which occurred after ~10 s since the subject started holding his breath. Then, as the subject returned to normal breathing, the PWV also returned to values that are similar to those measured in the duration before the subject held his breath. The result demonstrates that the proposed sensor can continuously measure the PWV for each pulse and is therefore suitable for continuous measurement of the blood pressure by measuring the PWV. In principle, it is possible to fabricate two cantilevers for the measurement of pulse wave on the same sensor chip whose size can be as small as several millimeters. This advantage allows the realization of ring-type and bracelet-type wearable devices for the PWV measurement using the proposed method.

## 4. Conclusions

In this study, we proposed and realized a sensor based on a MEMS piezoresistive cantilever to measure pulse waves. In recent years, a piezoresistive cantilever has been developed for highly sensitive measurements of force, pressure, and airflow. In the proposed sensor, the cantilever was placed inside an air chamber with a thin membrane that can be deformed by a pulse when in contact with the skin above a vessel. The deformation of the membrane results in pressure change in the chamber, which can be measured using the cantilever. In comparison with previous MEMS-based piezoresistive sensors in which the sensing elements were embedded inside elastomer, gel and liquid, the proposed sensor has the advantages of high sensitivity and excellent robustness. Moreover, in comparison to polymer-based sensor, the sensor in this study has an advantage of compatibility for mass-production because the fabrication process for the cantilever has been well-established. We showed that the sensor can measure pulse waves when attached to the skin above the vessel. Moreover, we demonstrated that the PWV for each pulse can be measured using two fabricated sensors. The proposed sensor will be useful in many healthcare applications such as continuous measurements of pulse waves, pulse wave velocity, and blood pressure.

## Figures and Tables

**Table 1:** Comparison of the proposed sensor with other devices proposed in previous studies.

	Principle	Sensitivity	Robustness	Portability	Effect of Skin Tone	Mass-Production Compatibility
Pulse oximeter [10,11]	Photoplethysmography-based	Fair	Good	Good	Yes	Fair
Polymer-based [14,15]	Force detection-based	Good	Good	Good	No	Poor
MEMS structure in elastomer, gel or liquid [18,19,20]	Force detection-based	Fair	Fair	Good	No	Good
This work	Force detection-based	Good	Good	Good	No	Good
